# Maternal syphilis treatment and pregnancy outcomes: a retrospective study in Jiangxi Province, China

**DOI:** 10.1186/s12884-020-03314-y

**Published:** 2020-10-27

**Authors:** Zhihua Wan, Huan Zhang, Haigang Xu, Yang Hu, Cai Tan, Yuling Tao

**Affiliations:** Department of Health Care, Jiangxi Provincial Maternal and Child Health Hospital, No. 318 Bayi Street, Nanchang, 330006 Jiangxi China

**Keywords:** Syphilis, Maternal treatment, Congenital syphilis, Pregnancy outcomes

## Abstract

**Background:**

Studies investigating the associations of maternal syphilis treatment with birth outcomes mainly concentrated in economically developed areas. Limited data are available in economically underdeveloped areas, such as Jiangxi Province. The study aims to investigate the impact of maternal treatment on birth outcomes in Jiangxi Province, China.

**Methods:**

Data were obtained from the China’s Information System of Prevention of Mother-to-Child Transmission in Jiangxi Province. All syphilis infected pregnant women who delivered ≥28 gestational weeks and were registered in this system between 1 January 2013 and 31 December 2019 were enrolled. Pregnancy outcomes were evaluated by group-specific analyses according to their treatment status, adequacy and initiation time.

**Results:**

4210 syphilis infected pregnant women were included in the analyses. Infants born to untreated mothers (*n* = 1364) were at significantly higher risk for stillbirth (adjusted odds ratio (aOR) = 1.74, 95% CI, 1.01–3.00, *P* = 0.045), preterm birth (aOR = 1.27, 95% CI, 1.02–1.59, *P* = 0.034) and low birth weight (LBW) (aOR = 1.44; 95% CI, 1.11–1.86, *P* = 0.006) than those born to treated mothers (*n* = 2846) after adjustment for confounding factors. A significantly higher risk of stillbirth (aOR = 3.68; 95% CI, 1.62–8.34, *P* = 0.002), preterm birth (aOR = 2.26; 95% CI, 1.71–3.00, *P* < 0.001), LBW (aOR = 2.23; 95% CI, 1.59–3.14, *P* < 0.001) and congenital syphilis (CS) (aOR = 3.63; 95% CI, 1.80–7.31, *P* < 0.001) was found in infants exposed to mothers treated inadequately (*n* = 1299) than those treated adequately (*n* = 1547). No pregnant women who initiated the treatment in the first trimester (*n* = 682) delivered a neonatal CS case. Compared with mothers who initiated treatment in the first trimester (*n* = 682), those initiated in the third trimester (*n* = 1234) suffered an increased risk of stillbirth (aOR = 4.48; 95% CI, 1.31–15.30, *P* = 0.017), preterm birth (aOR = 2.34; 95% CI, 1.61–3.40, *P* < 0.001) and LBW (aOR = 3.25; 95% CI, 1.97–5.37, *P* < 0.001).

**Conclusions:**

Maternal treatment, especially early and adequate treatment, plays a crucial role in mitigating adverse pregnancy outcomes among syphilis infected women.

## Background

Syphilis is a sexually and vertically transmitted disease caused by the bacterium Treponema pallidum. Maternal syphilis remains a significant public health problem worldwide. According to the global epidemiologic data, there were an estimated 988,00 infected pregnant women in 2016 [1]. Untreated or inadequately treated maternal syphilis is associated with increased risk of adverse pregnancy outcomes, including early fetal deaths, stillbirths, neonatal death, preterm birth, low birth weight (LBW), and congenital syphilis (CS) [2, 3]. Globally, it was estimated that there were 355,000 adverse birth outcomes attributed to maternal syphilis [1]. As the recognized adverse pregnancy outcomes and public health problems, the World Health Organization (WHO) launched an initiative to eliminate CS by improving syphilis screening and treatment coverage of pregnant women to prevention mother to child transmission.

In China, the number of maternal syphilis and CS cases increased annually since the 1990s [4]. In 2013, data from the Chinese national surveillance system showed that 15,884 pregnant women with syphilis infection delivered [5]. The reported cases of CS increased from 468 in 2000 to 10,032 in 2013, the corresponding incidence rate of CS increased dramatically from 2.6 cases in 2000 to 69.9 cases in 2013 per 100,000 live births [6]. In order to decrease the MTCT rate of syphilis and strengthen the healthcare for infected mothers and their infants, China released the National Implementation Guidelines on Integrated Prevention of MTCT (iPMTCT) of HIV, syphilis, and Hepatitis B Virus (HBV) Programme in 2011 [5]. Jiangxi Province is located in the southeast of China, belongs to East China. In 2018, the number of resident population, live births were 46.47 million and 558,666, respectively. According to Jiangxi provincial surveillance data, the number of syphilis-seropositive pregnant women who delivered increased from 570 in 2013 to 891 in 2018. In 2011, Jiangxi province integrated the screening and treatment of maternal syphilis into the provincial iPMTCT program of HIV, syphilis and HBV.

Currently, several studies had investigated the associations of treatment during pregnancy with pregnancy outcomes among syphilis infected pregnant women [7–10], mainly concentrated in economically developed areas. Limited data are available to evaluate the influence of maternal treatment on pregnancy outcomes in economically underdeveloped areas, such as Jiangxi Province. Furthermore, much of our knowledge regarding on the effectiveness of treatment to improve pregnancy outcomes in pregnancy comes from small observational studies or from the pre-penicillin era [3, 11]. The purpose of this study was to investigate the impact of maternal treatment during pregnancy on pregnancy outcomes in Jiangxi province.

## Methods

### Study design and participants

Data were obtained from the China’s Information System of PMTCT of Syphilis Management in Jiangxi Province. This system has been used to monitor and evaluate the prevalence of maternal syphilis and congenital syphilis in China. Surveillance of maternal syphilis is conducted through mandatory case-reporting by all health facilities providing delivery services. Detail information about this system was available elsewhere [5]. The regional program was established in 2011. During 2011–2013, the program was performed in 17 pilot counties. From 2013, this program was carried out throughout the whole province, and intervention services of maternal syphilis were offered for free in 40 counties. Since 2015, comprehensive intervention services of syphilis were offered to all pregnant women for free in the whole province, including syphilis counseling and testing at the first antenatal care, treatment for positive cases during pregnancy, follow-up service for exposed infants until they were diagnosed or excluded CS, etc. In this program, all pregnant women were screened with the non-treponemal test (rapid plasma regain (RPR) or toluidine red unheated serum test (TRUST)) or treponemal test (treponemal pallidum particle agglutination (TPPA) or enzyme linked immunosorbent assay (ELISA). Samples positive for screening were confirmed by the other test. A diagnosis of maternal syphilis infection was made when one had positive results in rapid plasma regain (RPR)/toluidine red unheated serum test (TRUST) and treponemal pallidum particle agglutination (TPPA)/enzyme linked immunosorbent assay (ELISA) test. Given that data which included pregnant women with early fetal loss and miscarriage was not available, only syphilis infected pregnant women who delivered at gestational age of 28 weeks or more (including live births, stillbirth ≥28 gestational weeks and 0–7 days for neonatal deaths) and were registered in Information System of PMTCT of Syphilis Management between 1 January 2013 and 31 December 2019 were enrolled. According to treatment status during pregnancy, pregnant women were classified into treated group with at least one course of treatment and untreated group with no treatment. In the treatment group, women were further grouped into: treated adequately and treated inadequately, initiated treatment in the first (≤12 gestational weeks), second (13–27 gestational weeks) and third (≥28 gestational weeks) trimester, respectively.

### Definitions

Adequate treatment was defined as two completed courses of penicillin treatment with more than 2 weeks (appropriately 4 weeks) between the two courses, and treatment must have been provided at least 28 days prior to delivery or 4 weeks gestation. Non-penicillin treatment or treatment with fewer than two completed courses was defined as treated inadequately. Pregnancy outcomes involved in this study included stillbirth, preterm birth, low birth weight (LBW), birth defects, asphyxia, pneumonia, and neonatal CS. Gestational age was based on the interval between the date of last menstrual period and the date of delivery. Preterm delivery was defined as delivery before 37 completed weeks of gestation. LBW was defined as a birth weight less than 2500 g. The diagnostic criteria of neonatal CS in the study referred to Dou L et al. [5]

### Statistical analysis

Data were analyzed with the use of SPSS version 18.0 (SPSS, Chicago, IL). Continuous variables were presented as means and standard deviations (SD) and were analyzed by *t* test. Categorical variables were expressed as numbers and percentages and were by the χ^2^ test. The impact of maternal treatment during pregnancy on pregnancy outcomes were examined by the odds ratio (OR) and its 95% confidence interval (CI) using unconditional multivariate logistic regression analysis adjusting for potential confounding variables. For all analyses, *P* values lower than 0.05 were regarded as statistically significant.

### Ethical considerations

We removed personal information of mothers and infants from the database and only kept their identification numbers. All information was kept confidential.

## Results

**Fig. 1:**
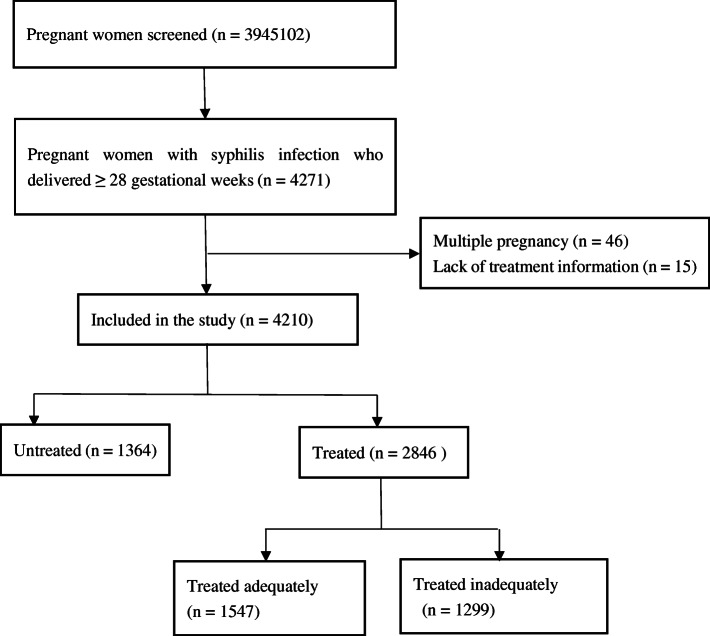
Flow chart of the study population. This figure demonstrates how the study population was selected

During 1 January 2013–31 December 2019, 3,945,102 pregnant women were screened for syphilis and 4271 who delivered at gestational age of 28 weeks or more and were registered in Information System of PMTCT of Syphilis Management of Jiangxi Province were enrolled. Among these women, 61 women were excluded from the study because of delivering multiple pregnancy (46) or lacking of treatment information (15), thus resulting in 4210 women included in the final analyses (Fig. 1). Characteristics of syphilis-seropositive pregnant women between the treated and untreated group are shown in Table 1. Women in the treated group were younger than those in the untreated group (*P* < 0.001). Significant differences in education (*P* < 0.001), employment (*P* = 0.007), marital status (*P* = 0.028), nulliparas (*P* < 0.001), and history of syphilis infection (*P* < 0.001) were observed between the two groups. In addition, compared with women in the treated group, those in the untreated group had a smaller proportion of attending the first antenatal care in first trimester (28.6% vs. 41.0%; *P* < 0.001) and being in the latent stage (76.6% vs. 84.5%; *P* < 0.001).

**Table 1:** Characteristics of syphilis-seropositive pregnant women with respect to treatment status

Characteristics	Treated (*n* = 2846)	Untreated (*n* = 1364)	*P*
Age (y)	28.50 ± 6.00	29.23 ± 6.15	**< 0.001**
Ethnicity of Han	2745 (96.5)	1329 (97.4)	0.091
Education			**< 0.001**
Primary school and illiteracy	424 (14.9)	266 (19.5)	
Junior high school	1630 (57.3)	729 (53.4)	
Senior high school	521 (18.3)	243 (17.8)	
College and above	182 (6.4)	66 (4.8)	
Unknown	89 (3.1)	60 (4.4)	
Employment			**0.007**
Unemployed	1616 (56.8)	744 (54.5)	
Worker without regular pay	656 (23.0)	345 (25.3)	
Ordinary employee	188 (6.6)	62 (4.5)	
Other	386 (13.6)	213 (15.6)	
Marital status			**0.028**
Single	147 (5.2)	52 (3.8)	
First marriage	2295 (80.6)	1144 (83.9)	
Other	404 (14.2)	168 (12.3)	
Nulliparas	872 (30.6)	214 (15.7)	**< 0.001**
History of syphilis infection	1091 (38.3)	397 (29.1)	**< 0.001**
Time of the first antenatal care			**< 0.001**
First trimester	1166 (41.0)	390 (28.6)	
Second trimester	842 (29.6)	218 (16.0)	
Third trimester	592 (20.8)	492 (36.1)	
Unknown	246 (8.6)	264 (19.4)	
Maternal syphilis stage			**< 0.001**
Latent stage	2406 (84.5)	1041 (76.6)	
Primary stage	120 (4.2)	40 (2.9)	
Secondary stage	22 (0.8)	7 (0.5)	
Tertiary stage	12 (0.4)	9 (0.7)	
Unknown	286 (10.6)	267 (19.6)	
Nontreponemal serum test titer			**0.019**
< 1:8	2022 (71.0)	968 (71.0)	
≥ 1:8	804 (28.3)	374 (27.4)	
Unknown	20 (0.7)	22 (1.6)	

Data are expressed as mean ± SD or number (percentage)

Significant values are in bold font

When pregnancy outcomes were analyzed according to treatment status, the infants born to untreated mothers were at significantly higher risk for stillbirth (aOR = 1.74, 95% CI, 1.01–3.00, *P* = 0.045), preterm birth (aOR = 1.27, 95% CI, 1.02–1.59, *P* = 0.034) and LBW (aOR = 1.44; 95% CI, 1.11–1.86, *P* = 0.006) than those born to the treated mothers after adjustment for maternal age, education, employment, marital status and parity (Table 2).

**Table 2:** Pregnancy outcomes of syphilis-seropositive pregnant women with respect to treatment status

Pregnancy outcomes	Treated^a^ (*n* = 2846)	Untreated (*n* = 1364)	OR (95% CI)^b^	*P*
Stillbirth	32 (1.1)	25 (1.8)	**1.74 (1.01–3.00)**	**0.045**
Preterm birth	238 (8.4)	143 (10.5)	**1.27 (1.02–1.59)**	**0.034**
Low birth weight	161 (5.7)	107 (7.8)	**1.44 (1.11–1.86)**	**0.006**
Birth defects	12 (0.4)	7 (0.5)	1.22 (0.47–3.16)	0.686
Asphyxia	47 (1.7)	23 (1.7)	0.99 (0.59–1.65)	0.973
Pneumonia	14 (0.5)	8 (0.6)	1.16 (0.48–2.82)	0.749
Neonatal CS	45 (1.6)	30 (2.3)	1.39 (0.86–2.25)	0.176

*OR* Odds ratio, *CI* Confidence interval, *CS* Congenital syphilis

Significant values are in bold font

^a^ Reference group

^b^ Adjusted for age, education, employment, marital status and parity

To further investigate the influence of adequate treatment and initiation time of treatment during pregnancy on pregnancy outcomes, 2846 treated women were included in the analysis. We found that a significantly higher risk of stillbirth (aOR = 3.68; 95% CI, 1.62–8.34, *P* = 0.002), preterm birth (aOR = 2.26; 95% CI, 1.71–3.00, *P* < 0.001), LBW (aOR = 2.23; 95% CI, 1.59–3.14, *P* < 0.001) and CS (aOR = 3.63; 95% CI, 1.80–7.31, *P* < 0.001) in infants born to mothers treated inadequately than those treated adequately after adjusting for maternal age, education, employment, marital status and parity (Table 3). Compared with infants exposed to mothers who initiated treatment in the first trimester, those who initiated treatment in the third trimester had an increased risk of stillbirth (aOR = 4.48; 95% CI, 1.31–15.30, *P* = 0.017), preterm birth (aOR = 2.34; 95% CI, 1.61–3.40, *P* < 0.001) and LBW (aOR = 3.25; 95% CI, 1.97–5.37, *P* < 0.001) after adjustment for maternal age, education, employment, marital status and parity. There were no infants diagnosed with CS from mothers who initiated treatment in the first trimester (Table 4).

**Table 3:** Pregnancy outcomes with respect to adequate treatment status among treatment group

Pregnancy outcomes	Treated adequately^a^ (*n* = 1547)	Treated inadequately (*n* = 1299)	OR (95% CI)^b^	*P*
Stillbirth	8 (0.5)	24 (1.8)	**3.68 (1.62–8.34)**	**0.002**
Preterm birth	86 (5.6)	152 (11.7)	**2.26 (1.71–3.00)**	**< 0.001**
Low birth weight	58 (3.7)	103 (7.9)	**2.23 (1.59–3.14)**	**< 0.001**
Birth defects	6 (0.4)	6 (0.5)	1.09 (0.34–3.49)	0.889
Asphyxia	21 (1.4)	26 (2.0)	1.46 (0.81–2.64)	0.210
Pneumonia	8 (0.5)	6 (0.5)	0.82 (0.28–2.44)	0.719
Neonatal CS	11 (0.7)	34 (2.7)	**3.63 (1.80–7.31)**	**< 0.001**

*OR* Odds ratio, *CI* Confidence interval, *CS* Congenital syphilis

Significant values are in bold font

^a^ Reference group

^b^ Adjusted for age, education, employment, marital status and parity

**Table 4:** Pregnancy outcomes with respect to initiation time of treatment among treatment group

Group	Stillbirth	Preterm birth	Low birth weight	Birth defects	Asphyxia	Pneumonia	Neonatal CS
Initiated treatment in the first trimester (≤12 weeks)^a^	3 (0.4)	40 (5.9)	20 (2.9)	1 (0.1)	8 (1.2)	5 (0.7)	0 (0)
Initiated treatment in the second trimester (13 weeks - 27 weeks)	6 (0.6)	48 (5.2)	37 (4.0)	6 (0.6)	13 (1.4)	4 (0.4)	11 (1.2)
OR (95% CI)^b^	1.41 (0.35–5.69)	0.89 (0.58–1.37)	1.35 (0.78–2.36)	4.24 (0.51–35.39)	1.20 (0.49–2.92)	0.57 (0.15–2.13)	**/**
P	0.634	0.588	0.287	0.183	0.685	0.399	**/**
Initiated treatment in the third trimester (≥28 weeks)	23 (1.9)	150 (12.2)	104 (8.4)	5 (0.4)	26 (2.1)	5 (0.4)	34 (2.8)
OR (95% CI)^b^	**4.48 (1.31–15.30)**	**2.34 (1.61–3.40)**	**3.25 (1.97–5.37)**	2.48 (0.28–21.86)	1.84 (0.81–4.17)	0.46 (0.13–1.69)	**2.26 (1.11–4.61)**^**c**^
P	**0.017**	**< 0.001**	**< 0.001**	0.413	0.142	0.243	**0.025**
P for trend	**0.003**	**< 0.001**	**< 0.001**	0.622	0.109	0.252	**< 0.001**

*OR* Odds ratio, *CI* Confidence interval, *CS* Congenital syphilis.

Significant values are in bold font

^a^ Reference group

^b^ Adjusted for age, education, employment, marital status and parity

^c^ Initiated treatment in the second trimester as reference group

To clarify whether initiation time of treatment influenced its adequacy, we performed a further analysis and found that significantly higher rates of adequacy when treatment was initiated in the first (84.8%) and second trimester (79.5%) than in the third trimester (18.6%) (*P* < 0.001) (Table 5).

**Table 5:** Adequate treatment associated with initiation time

Initiation time of treatment	Treated adequately	Treated inadequately	*P*
Initiated treatment in the first trimester (≤12 weeks)	578 (84.8)	104 (15.2)	**< 0.001**
Initiated treatment in the second trimester (13 weeks–27 weeks)	739 (79.5)	191 (20.5)	
Initiated treatment in the third trimester (≥28 weeks)	230 (18.6)	1004 (81.4)	

Significant values are in bold font

## Discussion

Our study showed that several characteristics among pregnant women regarding of treatment status distributed significantly differently, including maternal age, education, employment, marital status, parity, history of syphilis infection, time of the first antenatal care, syphilis stage and nontreponemal serum test titer. Fu-Chang Hong et al. [8] investigated factors related to non-treatment for maternal syphilis and found women who were less educated and had their first antenatal care at 28 gestational weeks or later were more likely to reject or miss treatment during pregnancy, which is in accordance with our study. Women with low educational level often lack knowledge about health care and at a low socioeconomic level. Therefore, a large proportion of those women remained untreated. Patient education should be strengthened to increased awareness of syphilis infection and willingness for treatment among women with poor education. Early antenatal care can facilitate early detection of maternal syphilis and prompt treatment for those with positive test [12]. In our study, the proportions of pregnant women who initiated their first antenatal care in the first two trimesters were much higher in the treated group than that in the untreated group (70.6% vs. 44.6%), highlighting the importance of initiation time of the first antenatal care on maternal syphilis treatment.

Studies regarding the influence of maternal syphilis treatment on pregnancy outcomes found that non-treatment was associated with increased risk of a series of adverse pregnancy outcomes, including stillbirth, preterm birth and LBW [2, 3, 13, 14], which is consistent with our finding. Treponema pallidum can spread to different organs of the fetus and cause damage to both placenta and umbilical cord from gestational week 16, resulting in stillbirth or premature birth by compromising fetal growth and viability [3]. Based on the China’s iPMTCT guidelines, all pregnant women could receive treatment for free as soon as a diagnosis of syphilis infection was made during pregnancy [5]. However, nearly one third of syphilis infected pregnant women in the current study remained untreated, which posed great risk for stillbirth, preterm birth and LBW. Hence, treatment in pregnancy should be reinforced for purpose of improving the pregnancy outcomes among pregnant women with syphilis infection.

Early diagnosis and early sufficient penicillin treatment play a crucial role on syphilis management in pregnancy [15–18]. In order to prevent CS and other adverse pregnancy outcomes caused by maternal syphilis, WHO recommends that all syphilis infected pregnant women should receive at least one dose of benzathine penicillin G (BPG) in the first trimester [19, 20]. The U.S. CDC guidelines recommend that maternal treatment should initiate as early in pregnancy as possible and comply with the recommended regimen per stage of syphilis [16]. China guidelines suggest at least two courses of penicillin treatment, one at early pregnancy and the other at the third trimester [5]. In our study, we observed that inadequate treatment and treatment initiated in late pregnancy remained risk factors for stillbirth, preterm birth, low birth weight and neonatal CS, which is similar with results obtained by other studies [7, 8, 21]. Notably, Qin JB et al. observed that every week of delay in treatment was related to 2.82-fold increased risk for adverse pregnancy outcomes [22]. In addition, we found that no pregnant women who initiated the treatment in the first trimester delivered a neonatal CS case, which was also observed by Xue Zhang et al. [9], adding more evidence on the essential effect of early treatment on preventing neonatal CS. Our study demonstrated that the proportion of adequate treatment decreased along with the initiation time of treatment. Taken together, the results of this study indicated that early treatment should be strengthened for the sake of minimizing the impact of maternal syphilis on pregnancy outcomes through promoting the treatment adequacy.

Our study had several limitations. First, the data were extracted from the China’s Information System of PMTCT of Syphilis Management in Jiangxi Province, which is a passive surveillance system. Therefore, the results of this study might be biased by underdetection, underreporting and misclassification. Second, CS might be underestimated in the study due to the inclusion of neonatal CS cases rather than all CS cases diagnosed any time up to 18 months of age. Third, only women who delivered ≥28 gestational weeks were included in the study. Hence, some adverse birth outcomes including early fetal loss and miscarriage might be underestimated. Fourth, comparison about pregnancy outcomes could not be done between women with and without syphilis infection owing to the lack of data availability. Further research regarding of the impact of maternal syphilis on pregnancy outcomes is warranted.

## Conclusions

This is the first study investigating the impact of maternal syphilis treatment during pregnancy on pregnancy outcomes in Jiangxi Province. The study suggested that treatment, especially early and adequate treatment, plays a crucial role in mitigating adverse pregnancy outcomes, such as stillbirth, preterm birth, LBW and neonatal CS. Regarding the fact that a large proportion of pregnant women with syphilis infection were untreated or inadequately treated or treated late in pregnancy in Jiangxi Province, strategies ensuring early screening, timely diagnosis, and early treatment should be reinforced in the future in order to improve pregnancy outcomes and promote the health of offspring.

## Data Availability

The datasets generated and/or analysed during the current study are not publicly available due involving personal privacy but are available from the corresponding author on reasonable request.

## Abbreviations

MTCT: Mother-to-child transmission
OR: Odds ratio
LBW: Low birth weight
CS: Congenital syphilis
HBV: Hepatitis B virus
RPR: Rapid plasma regain
TRUST: Toluidine red unheated serum test
TPPA: Treponemal pallidum particle agglutination
ELISA: Enzyme linked immunosorbent assay
SD: Standard deviation
CI: Confidence interval
